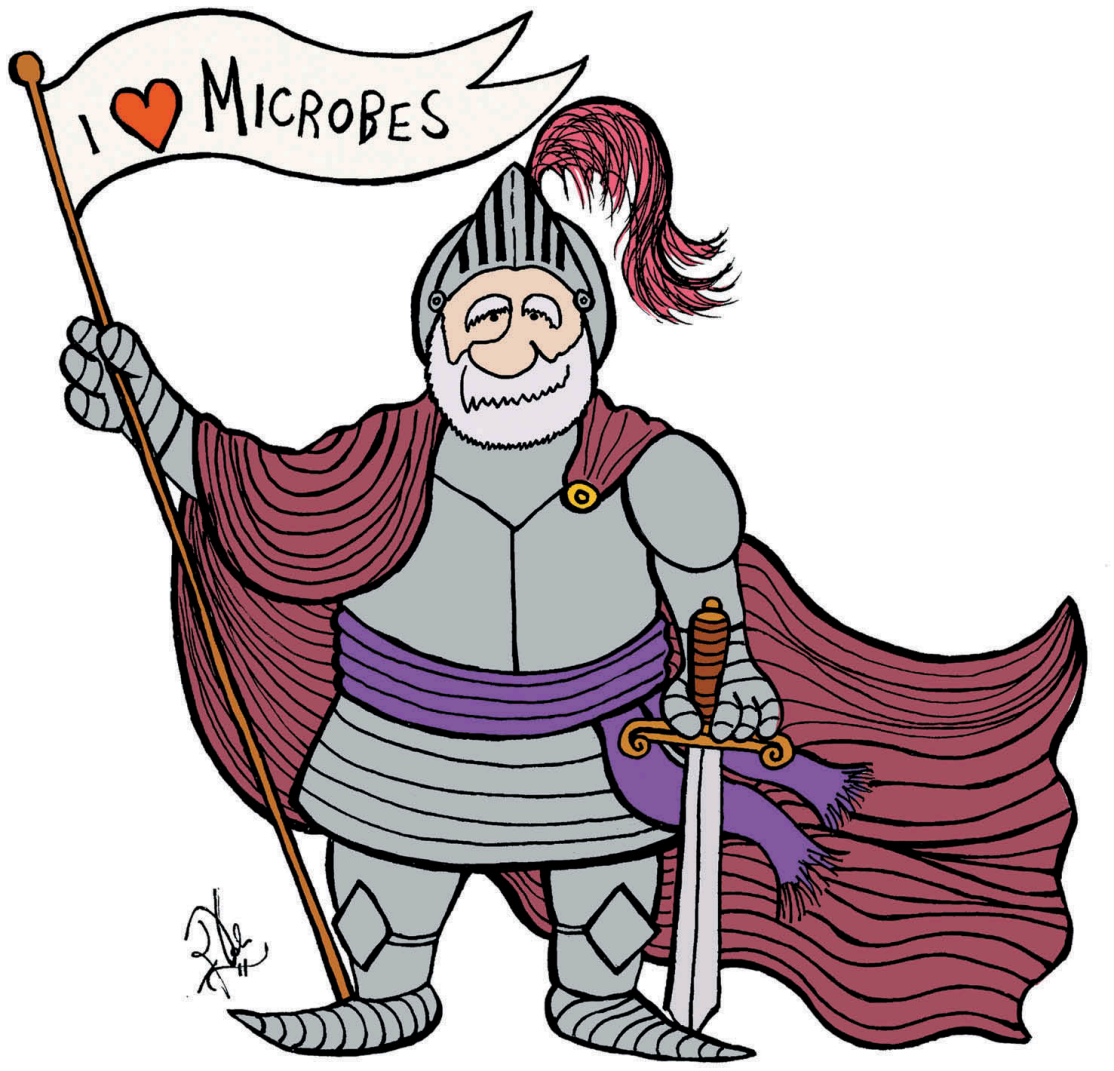# Sir Tore Midtvedt

**DOI:** 10.1080/16512235.2018.1535826

**Published:** 2018-11-08

**Authors:** Jørgen Valeur

**Affiliations:** Unger-Vetlesen Institute, Lovisenberg Diaconal Hospital, OsloNO-0440, Norway

Tore Midtvedt, former Editor-in-Chief of *Microbial Ecology in Health and Disease*, has been appointed Knight First Class of the Norwegian Royal Order of St. Olav. Midtvedt received the award on 8 October 2018 for his outstanding achievements in medical microbiology.

Midtvedt studied medicine in Oslo (1952–1956) and Bergen (1956–1958) at a time when the intestinal microflora was still a misunderstood and neglected organ, learning that its function was merely to modulate stool consistency. Fortunately, Midtvedt soon recognized that the microbes exert functions extending far beyond modifying the appearance of feces [1] – activities that he has carefully studied ever since, for more than half a century. His strategy to identify microbial functions has centered on a succinct equation [2]:

With a slight modification of terms first used by Claude Bernard, the mammalian organism itself or the host’s side of the ecosystem can be defined as *milieu interieur*, the nonhost side as *milieu exterieur*, and *milieu interieur* and *milieu exterieur* together as *milieu total*. […] A simple equation – *milieu total* minus *milieu interieur* gives *milieu exterieur* – or what the microbes have done.

Midtvedt earned his PhD degree at Karolinska Institutet in Stockholm in 1968. He held different positions at the University of Oslo until he was appointed Professor of Medical Microbiology in 1982. In 1983 he was appointed Professor and Chairman of the Department of Medical Microbial Ecology, Cell and Molecular Biology at Karolinska Institutet in Stockholm. He held this position until his retirement in 1999. In 2010 Midtvedt was promoted to Doctor of Veterinary Medicine Honoris Causa by the Faculty of Veterinary Medicine of the Norwegian University of Life Sciences. Midtvedt has supervised more than 50 PhD candidates, acted as opponent of doctoral dissertations in six countries, and published numerous scientific papers, abstracts, book chapters and reports. He is still involved in many research projects.

The journal would like to congratulate its former Editor-in-Chief for receiving the noble recognition from the King of Norway. The microbes would probably like to congratulate as well: they have all reasons to celebrate that their good old friend – who has always guarded them as a holy grail – finally has become a true knight!

**Figure:**